# The Prevalence of Chronic Pain in the Adult Population in Israel: An Internet-Based Survey

**DOI:** 10.1155/2022/3903720

**Published:** 2022-09-20

**Authors:** Haggai Sharon, Hila Greener, Uri Hochberg, Silviu Brill

**Affiliations:** ^1^Institute of Pain Medicine, Department of Anesthesia and Critical Care Medicine, Tel Aviv Sourasky Medical Center, Tel Aviv, Israel; ^2^Sagol Brain Institute, Wohl Institute for Advanced Imaging, Tel Aviv Sourasky Medical Center, Tel Aviv, Israel; ^3^Sackler Faculty of Medicine, Tel Aviv University, Tel Aviv, Israel; ^4^Sagol School of Neuroscience, Tel Aviv University, Tel Aviv, Israel

## Abstract

**Background:**

Chronic pain (CP) prevalence in different studies has been inconsistent, ranging from 12% in Spain to 42% in the UK.

**Purpose:**

We conducted an internet-based survey in a representative cohort of Israeli adults assembled by a large professional survey company in order to probe the prevalence of CP in Israel.

**Methods:**

8,300 Israeli adults comprising a representative cohort of the Israeli population were asked whether they were suffering from pain lasting over 3 months. 1647 participants responded (19.8% response rate). Of these, 515 (31.3%) had CP. Participants with CP were then asked a series of follow-up questions regarding their chronic pain. Statistical weights were used to correct for the distribution of the Israeli population based on sociodemographic characteristics.

**Results:**

CP patients were significantly older than respondents without pain. The average daily pain was 5.8/10 on a numerical rating scale. Common pain locations were axial skeleton and headaches. However, over half of patients reported pain in multiple body areas, and around a fifth had an undiagnosed chronic pain syndrome. Around 40% of pain patients reported to have visited a specialized pain clinic, and the same proportion has consulted several specialists. Despite this, a sizable proportion of high pain intensity patients were still left with no or inefficient treatment to alleviate their pain.

**Conclusions:**

This is the first internet survey conducted in Israel to estimate the incidence of CP, and the high CP prevalence documented is in agreement with previous reports from Europe and the USA. It also reaffirms the widespread existence of multifocal or widespread pain in clinical chronic pain and the correlation between pain intensity, impact on patients' quality of life and disability, and pain intractability. These data reaffirm the similarly major health burden CP presents across different countries and cultures.

## 1. Introduction

Chronic pain is a major global health problem and one of the most frequent reasons for seeking medical care [1–3]. Moreover, international clinical guidelines, as well as political statements and resolutions, view access to adequate pain therapy as a basic human right [4]. Unfortunately, even in countries with advanced health systems, chronic pain remains untreated and under-recognized in a sizeable proportion of the population.

Improving clinical pain management from a systems point of view first requires obtaining accurate prevalence estimates. Evaluating the burden of chronic pain in specific countries and communities is therefore essential in locally planning and allocating adequate socioeconomic resources to tackle the problem. This effort becomes crucial since published point-prevalence estimates of chronic pain from specific population-based surveys vary widely. The overall prevalence of persistent pain among adult primary care patients in 15 countries was estimated at 22% on average, but ranged from 5 to 33%. In Europe, for example, this varied from 12% in Spain to 30% in Norway [5]. Of note, this 2006 study, while including Israel, did not include any inferential statistics. In Japan, it has been suggested to be as high as 39.3% [6] and even a surprising 43.5% in the UK [7]. More recent population-based surveys in various countries, however, have more consistently estimated that 25-35% of adults report chronic pain [8]. A study by the US Centers for Disease Control and Prevention (CDC) estimated the point prevalence at 20.4% [9, 10]. Interestingly, a recent study has found 10-15% prevalence for chronic widespread pain in the adult US population [11]. Regardless of these variations in findings, it is clear that chronic pain is a prevalent condition across different countries, cultures, and geographic categories.

Such variation in prevalence estimates of chronic pain can be attributed, among other things, to differences in definitions of chronic pain, in types of populations studied, and in survey methodology. For example, currently, the International Association for the Study of Pain (IASP) defines pain as chronic if it persists beyond the normal tissue healing time (usually 3 months) [12], but there is no universally accepted standard definition for chronic pain.

In Israel, a large telephone survey in 2006 [5], almost one in seven surveyed Israeli adults reported having moderate or severe chronic pain, defined as pain lasting at least 6 months and with moderate-to-severe pain being experienced in the last month and at least twice a week. A similar large random survey was performed among patients from the largest healthcare insurer and provider in Israel in 2008 [13] that found an overwhelming 46% prevalence of chronic pain in at least one body area. Women suffered significantly more than men, as did those who were older, less educated, and born in Israel and Eastern Europe. Only 4.8% of the patients suffering from chronic pain were referred to pain specialists and 11% used complementary medicine.

In the current study, we aimed to re-explore the prevalence and clinical attributes of chronic pain in the adult Israeli population in light of a few important changes which occurred since these were last evaluated. First, we wished to assess pain prevalence according to the current IASP definition of chronic pain. Second, pain management has become a recognized and board-certified subspecialty in Israel in 2010, supporting the widespread establishment of dedicated pain services in Israel [14], which were far less prevalent prior to 2010. Lastly, and different from previous surveys, in order to achieve this aim, we conducted a web-based population-based survey in a representative sample of Israeli adults using an Internet-administered survey rather than an interviewer- conducted telephone questionnaire. This was not readily available in 2008 and allows for more undirected and unbiased sampling of respondents.

## 2. Methods

### 2.1. Study Population and Samples and Data Collection

The current point-prevalence survey was conducted by randomly distributing an Internet questionnaire to individuals included in a panel representative of the Israeli population. The panel is recruited and maintained by the Israeli survey research company MIDGAM, which provides national representative sample online data collection services in Israel. The survey was conducted and data were collected during June 2017. Participants were recruited from the company's existing large-scale online panel pool (over 100,000 members).

As the panel is limited to web users, statistical weights were used to correct the distribution of responders to the distribution of Israeli population based on sociodemographic characteristics and to account for survey nonresponse. The sample included all Israeli citizens aged 18 or older. The study referred to the following two, partly overlapping, samples: a major population—all Israeli citizens aged 18+, and subpopulation—individuals who report suffering from chronic pain. The sample of the major population (*n* = 1647) was drawn by strata sampling, and the subsample (*n* = 515) was filtered out from the major sample. The sample size of 500 was determined as the minimal target recruitment number to reach a confidence interval of 95% and a maximal sampling error of 4.4%. All responders aged 18 years or older filled a sociodemographic questionnaire and were then asked whether they suffer from “any kind of chronic pain that is constant or recurrent, for 3 months or longer”. If they answered positively, they filled a “pain questionnaire.”

Review and approval by an IRB were not deemed necessary due to the following considerations: 1. It was a web-based study targeting a general (i.e., not specifically clinical) population, with no direct measurement of biological properties and not involving any medical interventions; 2. All respondents belong to a registered cohort of a professional survey company and have agreed to have responses documented and published when joining the cohort; 3. Responses were completely anonymized, and no identifiable personal data regarding the responders were collected in any data base.

### 2.2. Statistical Analysis

Data analysis was conducted according to the following stages:

1. Applying statistical weights to correct biases in sociodemographic profile of major population/
2. Analysis of the dependent variables in both samples.
3. Association between independent and the dependent variables. The association between each of the independent and each of the dependent variables was tested by Chi-square or analysis of variance; the association between series of independent variables and a dependent variable was carried out by Pearson correlation (for quantitative dependent variable).

## 3. Results

An initial approach questionnaire was sent to 8,300 individuals included in the panel and 1,647 (19.8%) responded, 914 women (55.5%) and 733 (44.5%) men. Presented is the point-prevalence weighted for the Israeli population demographic characteristics with CI = 95%. Of the responders, 515 individuals (31.3%) reported suffering from chronic pain (CP) according to the questionnaire definition.

### 3.1. Sociodemographic Characteristics of Individuals with or without CP

Table 1 describes the sociodemographic characteristics of patients with chronic pain (CP) compared to those without CP.

**Figure 1:**
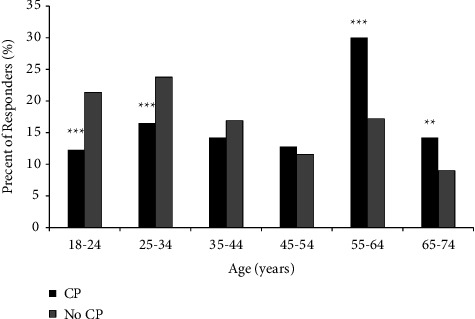
Responders with CP as a percentage of total responders in each group age.

The mean and median age of responders suffering from CP were significantly older compared to responders without CP (mean: 46.55 ± 15.8 vs. 40.01 ± 15.6, *p* < 0.001 respectively, and median 49.0 vs. 36.7, *p* < 0.001, respectively) (Figure 1). The prevalence of women in the group that reported CP was significantly higher than in the group without CP (59.8% vs. 53.6%, *p* < 0.05, respectively) while the prevalence of men with CP was lower than those without CP (40.2% vs. 46.4%, *p* < 0.05, respectively). The prevalence of older people over 55 years among CP group was significantly higher than those without CP (30.0% vs. 17%, *p* < 0.001, respectively). The CP group had a significantly higher prevalence of retirees than those without CP (14.3% vs. 8.7%, *p* < 0.05, respectively) and a significantly lower prevalence of full-time employees (47.4% vs. 55.2%, *p* < 0.05, respectively).

### 3.2. Chronic Pain Characteristics

Table 2 describes chronic pain characteristics among CP responders. More than two thirds (68.5%) of the CP responders reported CP duration longer than a year, 28.5% of them suffer from CP longer than 6 years. Interestingly, this was not correlated with age. Daily or constant pain was reported by 50.1% of responders. The average pain intensity level (Visual Analog Scale, VAS) was 5.8 ± 2.0 with 73.6% of CP respondents reporting pain intensity level of 5 or above. The most prevalent pain sites were lower back (36.3%), head (21.3%), and neck (13.7%). However, 57.5% of the CP responders reported CP in more than a single body location. A fifth of the responders with CP replied they did not know the etiology for their pain.

Almost half (47.3%) of the CP responders consulted their physician regarding their pain, and 39.3% of the CP responders have visited a pain clinic. 39.5% of the responders with CP have consulted more than a single medical doctor.

Pain-relieving treatments were used by 66.1% of the CP responders. 33.7% of them used nonpharmacological therapies. 31.8% of CP responders used alternative medicine. The most frequent were acupuncture and massage (41.1% and 30.1%, respectively). Medical cannabis was used by 3.7% of the CP responders, most of them used it illicitly (58%) and only 36.3% had a permit for medical use. The medication type used for CP treatment was almost equally divided between individuals taking over-the-counter (OTC) medications only, prescription medications only, or concurrent use of both prescription and OTC medications (21%, 20.2%, and 25.2%) (Table 2).

### 3.3. Responders' Characteristics According to Chronic Pain Intensity

In order to better detect trends and pain properties in the CP group, CP responders were grouped into three subgroups based on reported pain intensity (VAS): 1-3 VAS units (low intensity), 4-7 VAS units (moderate intensity), and 8-10 VAS units (high intensity) (Table 2). No statistically significant differences were found between the various pain intensity groups regarding mean age, sex, income, education levels, marital status, and religiosity/secularism selfdefinition.

Responders in the high-intensity pain group were less satisfied with their pain-relieving treatment: 20.4% reported no beneficial effect compared to moderate- and low-pain intensity groups (7.4% and 3.6%, respectively, *p* < 0.05). Nevertheless, only a minority (12.5%) of responders in the high-pain intensity group did not get any medical treatment compared to moderate- and low-pain intensity groups (34.5% and 59.9%, respectively, *p* < 0.05). Responders in the high-pain intensity group used more medications (both OTC and prescribed) than moderate- and low-pain intensity groups (37.8% vs. 22.9% and 17.4%, respectively, *p* < 0.05). In addition, they had a greater use of complementary treatments than the two other groups (43.2% vs. 32.1% and 15.3%, respectively, *p* < 0.05).

Responders in the high-pain intensity group were more likely to visit a pain clinic (57.8%) than those of moderate- and low-pain intensity groups (37.1% and 23.7%, respectively, *p* < 0.05). The major reasons for not using past medications or past treatment are lack of influence and high cost. Side effects accounted for 6.7% of medication discontinuation in the high-intensity pain group.

Responders in the high-intensity pain group were more likely to suffer from multiple medical conditions or illness causing their pain (42.2%) than the moderate- and low-pain intensity groups (29.2% and 16.9%, respectively, *p* < 0.05). They have also consulted more medical doctors regarding their pain (58.9% vs. 36.9% and 24% in the moderate- and low-pain intensity groups, respectively, *p* < 0.05).

Table 3 describes life quality as measured by the ability to perform routine tasks and to participate in social activities. Responders in the high-pain intensity group had more limitations or inability in performing daily physical activities and routine tasks such as walking, housework, lifting objects, exercising, and driving than those of moderate- and low-pain intensity groups (*p* < 0.05, Table 3). There was higher frequency of responders in the high-pain intensity group that were less capable of participating in social activities, meeting friends and family, working outside, and being autonomous than before compared to the other groups (*p* < 0.05, Table 3). Sleeping disturbances were also more common in the high-pain intensity group. Higher rate of them reported worse sleeping than before (44.7%) compared to the moderate- and low-frequency intensity groups (30.8% and 20.6%, respectively, *p* < 0.05). Overall quality of life was negatively correlated with pain intensity (*R* = −0.34, *p* < 0.01).

## 4. Discussion

Chronic pain exerts a substantial toll on patients and is a major public health concern [2, 15]. Estimating the prevalence of chronic pain in the population is a crucial first step for defining and addressing the needs for pain management services at the systems level, especially in countries with a centralized health care system such as Israel. The current pain prevalence survey is the third large-scale population survey conducted in Israel, and the first in over 12 years. Accordingly, it utilized a web-based approach to population sampling and employed a specialized survey company with experience in constructing a weighted sample representative of the Israeli population from a large existing cohort.

This is the first internet-based survey conducted in Israel to estimate the incidence of CP, and the prevalence found (31.7%) is in accordance with more recent studies of pain prevalence reported for Europe [8] and the USA [16] which was estimated to be between 25% and 35%. Nevertheless, large differences in CP prevalence exist between the current findings and those of older phone-based surveys previously conducted in Israel. However, there is already a large variation between the two phone-interview surveys conducted during 2006 (17%) [5] and 2008 (46%) [13]. This probably reflects the crucial effect the definition of CP has on the prevalence found in a specific survey, as was previously discussed in several papers. Thus, our prevalence is similar to that of 30.6% found in a USA-based Internet survey [16], and to that found in several European countries, reflecting comparable CP definition. Similarly, the higher (46%) and lower (17%) CP incidence found in Israel by Neville et al. [13] and Breivik et al. [5], respectively, most likely reflect more flexible or strict definitions, respectively, rather than an actual change in pain prevalence in Israel in those years. Indeed, when we limited our survey results to include only individuals reporting pain severity of ≥5, only 23.2% of our responders fit into this CP definition, a level closer to that found by Breivik et al. [5], which included similar duration and frequency restrictions.

Similar to previous studies in pain epidemiology, we found that patients with chronic pain were significantly older [11]. A larger proportion of them were either part time employed or retired. This may reflect the correlation with age or the result of medical disability resulting from chronic pain and other comorbidities. More than two thirds (68.5%) of the CP responders reported a CP duration longer than one year and daily or constant pain was reported by half of them. A vast majority reported pain intensity levels of 5 or above. In addition, around 40% of pain patients reported to have visited a specialized pain clinic and the same proportion has consulted several specialists regarding their pain, reflecting the high use of medical resources by patients with chronic pain. These findings indicate the severity of chronic pain problem both at the individual and at the societal level [17].

Common pain locations were axial skeleton and headaches, in agreement with current literature [18]. It is interesting to note, though, that over half of chronic pain patients have pain in multiple body areas, and that around a fifth had a chronic pain syndrome without a diagnosed underlying cause. These last two points serve to stress the complexity of clinical pain management, where there are often several organic pain generators or on the other side of the spectrum no identifiable one.

In contrast to previous surveys conducted in Israel and elsewhere, we did not find a significant sex difference in CP sufferers. In addition, we did not find the often described correlation between CP and education or income level. This may result from the sample size of the survey or may reflect a bias of selecting Internet users only, which leads to under-representation of people with no internet access that may belong to lower socioeconomic levels.

### 4.1. Predicting Variables and Correlations

To better characterize the heterogeneous group of CP sufferers, we divided it into subgroups based on reported pain intensity (Table 2). In accordance with previous surveys conducted in Spain [19] and Germany [20], the high (VAS 8-10) and moderate (VAS 4-7) pain intensity categories consisted a fifth and two thirds of the general CP group, respectively. Women tended to report more moderate and high intensity pain and consisted of 57-60% of the responders in these groups, similar to the findings described in previous studies.

Not surprisingly, a larger proportion of patients in the high-intensity pain subgroup visited pain clinics and used complementary and alternative medicine treatments. Despite this, they were overall less satisfied with their current treatment. In spite of this dissatisfaction, only 12.5% of responders with high-pain intensity did not receive any treatment at the time of the survey as opposed to 59.9% of the low-pain intensity group. In accordance with previous reports [9], there was also a significant negative correlation between pain intensity and daily activity, quality of life, and sleep measures. Over 50% of the responders with high-pain intensity were less able or unable to perform physical activities (walking, lifting, exercising, and housekeeping chores) and about 45% reported significantly disordered sleep. Around 40% of the responders in this group were less able or unable to attend social activities, work outside their home, or maintain satisfactory sexual relationships. These observations further stress the catastrophic impact chronic pain exerts of patients' lives and well-being. Similar results were found by Breivik et al. in 2006 in Europe [5] including Israel, indicating that CP effects are both common over a vast geographic and cultural range, but also that despite medical progress in the field of pain, its clinical repercussions at the population level have remained similar over the past 14 years. This is especially disappointing considering the fact that we found that a high percentage (40%) of CP patients were referred to pain specialists. Moreover, patients report that they have attempted large number of treatments and different medications, many of whom use multiple drugs and treatments concurrently, and many change treatments and medications occasionally. Despite all these, the most prevalent sentiment among patients regarding their pain treatment is that it is only partially helpful (47.5%), and 20% of patients suffering from chronic pain rate their treatment as unhelpful. In addition, a sizable proportion of high-pain intensity patients are still left with no or inefficient treatment to alleviate their pain (32.9% vs. 31% European mean for 2006 [5]).

### 4.2. Limitations

The current survey is smaller (though on the same scale) than the previous two surveys conducted in Israel, mainly because of low response rates, which are often seen in population-based Internet surveys. The survey methodology may have also impacted the results in that Internet-based surveys are prone to underestimation of actual chronic pain in the general population, as subpopulations known to have higher prevalence of CP (e.g., sick and elderly people, those in low socioeconomic levels, and those living in nursing homes) may be under-represented. Recruitment of Internet users only poses an additional selection bias of these populations. Finally, as the information does not come from medical records, but rather relies only on selfreports, it may not always be as reliable as objectively documented data registered in real time.

## 5. Conclusions

The current survey is the third to evaluate CP prevalence in Israel, the previous two conducted over a decade ago. Our results demonstrate the resemblance of CP prevalence and characteristics between Israel and recent surveys performed in other countries. Our results show that, compared to older surveys, a similar percentage of people suffering from moderate- and high-intensity pain report unsatisfactory pain relief from current treatments and reduced QOL indices, indicating that medical and technological advances made during this time remain insufficient to properly address chronic pain. There is, therefore, an urgent need to both develop better clinical approach to manage chronic pain and to optimize current chronic pain management at the systems level.

## Figures and Tables

**Table 1:** Demographic characteristics of responders with or without chronic pain.

	Responders with CP (*n* = 515)	Responders without CP (*n* = 1,131)	*p*-Value
**Females ** *n*(%)	308 (59.8%)	606 (53.6%)	*p* < 0.05

**Age n(%)**			
18-24	63 (12.3%)	242 (21.4%)	*p* < 0.001
25-34	85 (16.5%)	269 (23.8%)	*p* < 0.001
35-44	73 (14.2%)	192 (16.9%)	ns
45-54	66 (12.8%)	132 (11.6%)	ns
55-64	155 (30.0%)	195 (17.2%)	*p* < 0.001
65-74	73 (14.2%)	102(9.0%)	*p* < 0.01

**Mean age**	46.55 ± 15.8	40.01 ± 15.6	*p* < 0.001

**Median age**	49.0	36.7	*p* < 0.001

**Employment n(%)**			
Full time	244(47.4%)	624 (55.2%)	*p* < 0.01
Part time	111 (21.5%)	229 (20.3%)	ns
House keeping	13 (2.6%)	22 (2%)	ns
Retired	74 (14.3%)	98 (8.7%)	*p* < 0.001
Unemployed	35 (6.7%)	51 (4.5%)	ns
Other	38 (7.4%)	106 (9.4%)	ns

**Table 2:** Pain characteristics of three pain intensity groups.

VAS	Pain intensity			Total CP group	*p*-value
	1-3	4-7	8-10		
**Number of responders (%)**	*n* = 79 (15%)	*n* = 327(64%)	*n* = 109 (21%)	*n* = 515	ns
**Sex (%, n** **=** **515)**					
Male	50.5	37.8	39.8	44.5	ns
Female	49.5	62.2	60.2	55.5	ns

**Duration of pain (%, n** **=** **515)**					
6 months	12.2	10	9.1	10.1	ns
7-12 months	20.7	18	11.7	17.1	ns
1-3 years	22.5	23.4	21.1	22.8	ns
3-5 years	14.5	16.4	21.5	17.2	ns
6-10 years	22.6	27.8	34.9	28.5	ns
Do not know	7.5	4.4	1.6	4.3	ns

**Pain frequency (%, n** **=** **515)**					
Constant	16.9	18	27.7	19.9	ns
Daily	26.8	31.2	29.5	30.2	ns
At least once a week	30.4	31.0	20.2	28.6	ns
At least once a month	18.8	14.8	16.6	15.8	ns
Less than once a month	7.1	5	6	5.5	ns

**Pain locations (%, n** **=** **485)**					
One location	49.2	42	39.1	42.5	ns
Multiple locations	50.8	58	60.9	57.5	ns

**Satisfaction from pain treatment (%, n** **=** **485)**					
Very helpful	6.2	8.6	12.9	9.1	ns
Partially helpful	30.3	49.5	54.2	47.5	ns
Unhelpful	3.6	7.4	**20.4** ^*∗∗∗*^	9.5	*p* < 0.001^^^
Not receiving any treatment	59.9	34.5	**12.5** ^*∗∗∗*^	33.9	*p* < 0.001^^^

**Type of medications (%, n** **=** **485)**					
Only OTC medication	24.8	22.3	14.3	21	ns
Only prescribed medication	12.3	19.2	28.9	20.2	ns
Both OTC and prescribed medications	17.4	22.9	**37.8** ^*∗∗*^	25.2	*p* < 0.01^^^
No treatment based on medications	45.5	35.6	**19.0** ^*∗∗*^	33.7	*p* < 0.01^^^

**Current use of multiple prescribed medications (%, n** **=** **485)**	12.5	18.8	**32.5** ^*∗∗*^	20.7	*p* < 0.01^^^

**Use of complementary medicine (%, n** **=** **485)**	15.3	32.1	**43.2** ^*∗∗*^	31.8	*p* < 0.01^^^

**Current use of medical cannabis (%, n** **=** **485)**	3.5	3.3	5.3	3.7	ns

**Past use of medical cannabis (%, n** **=** **485)**	1.2	4.0	4.7	3.7	ns

**Reasons for not using previous medications (%, n** **=** **218)**					
Lack of effect	37.5	53.1	55.9	51.8	ns
Price	34.2	24.5	24.8	25.8	ns
CP improvement by more effective treatment	28.1	9.9	7.8	11.7	ns
Complicated treatment	10.9	8.3	8.3	8.6	ns
Too many medications	4	7.9	8.7	7.6	ns
Side effects	0.0	4.1	6.7	4.2	ns

**Consulting more than a single physician for CP (%, n** **=** **515)**	24	36.9	**58.9** ^*∗∗∗*^	39.5	*p* < 0.001^^^

**Visit a pain clinic (%, n** **=** **485)**	23.7	37.1	**57.8** ^*∗∗∗*^	39.3	*p* < 0.001^^^

^ between 8 and 10 pain intensity group and each of the other two groups.

**Table 3:** Life quality indices of pain intensity groups.

VAS	Pain intensity			Total CP group	*p*-value
	1-3	4-7	8-10		
Number of responders (%)	*n* = 75 (15%)	*n* = 309 (64%)	*n* = 101 (21%)	*n* = 485	
**Walking**					
Less able	16.3	23.1	**41.2** ^*∗∗∗*^	25.8	*p* < 0.001^^^
Unable	0.0	1.3	4.2	1.7	ns

**Lifting objects**					
Less able	29.5	48.1	50.4	45.7	ns
Unable	2.8	4.3	**17.3** ^*∗∗∗*^	6.8	*p* < 0.001^^^

**Exercise**					
Less able	43.3	46.5	41.9	45.1	ns
Unable	3.9	8.1	**26.3** ^*∗∗∗*^	11.2	*p* < 0.001^^^

**Housework**					
Less able	15.1	35.8	**51.5** ^*∗∗∗*^	35.8	*p* < 0.001^^^
Unable	0.0	1.2	5.8	2.0	ns

**Driving**					
Less able	1.2	7.0	**20.5** ^*∗∗*^	8.9	*p* < 0.01^^^
Unable	0.0	1.6	3.9	1.8	ns

**Social activities**					
Less able	6.1	17.5	**36.2** ^*∗∗∗*^	19.6	*p* < 0.001^^^
Unable	0.0	0.4	5.9	1.4	ns

**Working away from home**					
Less able	6.0	16.5	**34.0** ^*∗∗∗*^	18.5	*p* < 0.001^^^
Unable	1.4	3.6	10.0	4.6	ns

**Sleeping**					
Less able	20.6	30.8	**44.7** ^*∗∗*^	32.1	*p* < 0.01^^^
Unable	1.4	2.6	5.2	2.9	ns

**Having sexual relations**					
Less able	4.9	22.8	29.8	21.5	ns
Unable	2.8	1.9	**9.9** ^*∗∗∗*^	3.7	*p* < 0.001^^^

**Being independent**					
Less able	1.4	9.9	**27.3** ^*∗∗∗*^	12.2	*p* < 0.001^^^
Unable	0.0	0.5	1.0	0.6	ns

^between 8 and 10 pain intensity group and each of the other two groups.

## Data Availability

The registry data used to support the findings of this study are available from the corresponding author upon request subject to approval by the third party survey company.

## References

[1] Mäntyselkä P., Kumpusalo E., Ahonen R. (2001). Pain as a reason to visit the doctor: a study in Finnish primary health care. *Pain*.

[2] Rice A. S. C., Smith B. H., Blyth F. M. (2016). Pain and the global burden of disease. *Pain*.

[3] Arianna Camilloni G. N., Maggiolini P., Romanelli A. (2021). Chronic non-cancer pain in primary care: an Italian cross-sectional study. *Signa Vitae*.

[4] Cousins M. J., Lynch M. E. (Dec 2011). The Declaration Montreal: access to pain management is a fundamental human right. *Pain*.

[5] Breivik H., Collett B., Ventafridda V., Cohen R., Gallacher D. (May 2006). Survey of chronic pain in Europe: prevalence, impact on daily life, and treatment. *European Journal of Pain*.

[6] Inoue S., Kobayashi F., Nishihara M. (2015). Chronic pain in the Japanese community-prevalence, characteristics and impact on quality of life. *PLoS One*.

[7] Fayaz A., Croft P., Langford R. M., Donaldson L. J., Jones G. T. (2016). Prevalence of chronic pain in the UK: a systematic review and meta-analysis of population studies. *BMJ Open*.

[8] Breivik H., Eisenberg E., O’Brien T. (2013). The individual and societal burden of chronic pain in Europe: the case for strategic prioritisation and action to improve knowledge and availability of appropriate care. *BMC Public Health*.

[9] Dahlhamer J., Lucas J., Zelaya C. (2018). Prevalence of chronic pain and high-impact chronic pain among adults - United States, 2016. *MMWR. Morbidity and Mortality Weekly Report*.

[10] Yong R. J., Mullins P. M., Bhattacharyya N. (Feb 1 2022). Prevalence of chronic pain among adults in the United States. *Pain*.

[11] Mansfield K. E., Sim J., Jordan J. L., Jordan K. P. (2016). A systematic review and meta-analysis of the prevalence of chronic widespread pain in the general population. *Pain*.

[12] Treede R. D., Rief W., Barke A., Aziz Q. (2019). Chronic pain as a symptom or a disease: the IASP classification of chronic pain for the international classification of diseases (ICD-11). *Pain*.

[13] Neville A., Peleg R., Singer Y., Sherf M., Shvartzman P. (2008). Chronic pain: a population-based study. *The Israel Medical Association Journal: IMAJ*.

[14] Hochberg U., Sharon H., Bahir I., Brill S. (2021). Pain management—a decade’s perspective of a new subspecialty. *Journal of Pain Research*.

[15] Raffaeli W., Arnaudo E. (2017). Pain as a disease: an overview. *Journal of Pain Research*.

[16] Johannes C. B., Le T. K., Zhou X., Johnston J. A., Dworkin R. H. (2010). The prevalence of chronic pain in United States adults: results of an Internet-based survey. *The Journal of pain*.

[17] Cohen S. P., Vase L., Hooten W. M. (2021). Chronic pain: an update on burden, best practices, and new advances. *Lancet (London, England)*.

[18] St Sauver J. L., Warner D. O., Yawn B. P., Jacobson D. J. (2013). Why patients visit their doctors: assessing the most prevalent conditions in a defined American population. *Mayo Clinic Proceedings*.

[19] Pérez C., Margarit C., Serrano M. (Jun 2013). Survey of European patients assessing their own noncancer chronic pain: results from Spain. *Current Medical Research and Opinion*.

[20] Müller-Schwefe G. H. (2011). European survey of chronic pain patients: results for Germany. *Current Medical Research and opinion*.

